# Herpes Simplex Virus Causing Necrotizing Granulomatous Lymphadenitis

**DOI:** 10.7759/cureus.23709

**Published:** 2022-03-31

**Authors:** Vrajesh Parmar, Maha Bayya, Vivek Kak

**Affiliations:** 1 Internal Medicine, Henry Ford Health System, Jackson, USA; 2 Infectious Disease, Henry Ford Health System, Jackson, USA

**Keywords:** valacyclovir, chronic lymphocytic leukemia, viral inclusion bodies, herpes simplex virus, necrotizing granulomatous lymphadenitis

## Abstract

Localized necrotizing granulomatous lymphadenitis (GLA) is a very rare presentation of herpes simplex virus (HSV) infection. We are reporting a case that required multidisciplinary expertise to confirm the diagnosis and effectively treat the patient. Our patient had a recent diagnosis of chronic lymphocytic leukemia/small lymphocytic lymphoma (CLL/SLL) and presented with hematuria and palpable inguinal lymph nodes. Affirmative diagnosis required a core biopsy of the lymph node with immunochemistry staining and polymerase chain reaction (PCR) testing. This case reviews the unusual presentation of an HSV infection and emphasizes the importance of maintaining a high index of suspicion for infection when treating an immunocompromised patient with persistent symptoms.

## Introduction

A granuloma is a focal aggregate of immune cells that form in response to a persistent inflammatory stimulus. It demonstrates the compact organization of mature macrophages. Granulomas were first recognized as “tubercules” in the lungs of tuberculosis sufferers in early 1679 [1,2].

Granuloma formation begins with an inflammatory trigger, such as an infectious pathogen or a foreign body. As a part of the innate immune response, macrophages gather at the site of inflammation. An additional immune response may be activated if the activated macrophages are unable to control inflammatory stimulus efficiently. This additional immune response is orchestrated by dendritic cells and major histocompatibility complex II. Additional macrophages are recruited to the site, and a chronic inflammatory reaction develops. A peripheral layer of lymphocytes around the macrophages can form at the inflammatory stimulus. This inflammatory focus is known as granuloma. Maturing granuloma can develop necrosis. Infectious granulomas frequently become necrotic, while those caused by sarcoidosis and Crohn's disease generally do not, even when large and mature. Necrosis is generally thought to be a pathological process and is known to be associated with increased morbidity and transmission [3-5].

Infectious and noninfectious triggers can result in granuloma formation. Infectious stimuli include bacteria such as Mycobacteria, parasites such as Schistosoma, and fungi such as Aspergillus. Noninfectious triggers include autoimmune disease, neoplasms, and foreign bodies such as suture materials. In general, differential diagnosis of necrotizing granulomatous lymphadenitis (NGL) includes infectious etiology (with the most common being tuberculosis and fungal infection), malignant disorders, and high-grade lymphoid malignancies (Richter’s transformation); on the other hand, viral infection is an extremely uncommon cause of NGL, and few cases have been reported to be caused by HSV and classically associated with underlying chronic lymphocytic leukemia (CLL) [1].

Infectious lymphadenitis is a rare but important complication in CLL patients. An observational study done at Mayo Clinic Rochester included patients with CLL who had diagnostic lymph node biopsy to evaluate rapidly progressive or symptomatic lymphadenopathy. Only 3.9% of patients had infectious lymphadenopathy [6].

## Case presentation

Case report and diagnosis

A 73-year-old female with a past medical history of noninvasive melanoma status post resection, monoclonal (M) gammopathy with hypogammaglobulinemia (IgG) and recent diagnosis of chronic lymphocytic leukemia/small lymphocytic lymphoma (CLL/SLL), presented with hematuria and palpable left inguinal lymph nodes with workup showing inflammation and necrosis of left inguinal lymph nodes. Three months earlier, she complained of groin pain, hematuria, and weight loss with bilateral inguinal lymphadenopathy on the exam. A complete blood count (CBC) did not show any leukocytosis; the obtained computed tomography (CT) scan showed generalized retroperitoneal, mesenteric, and iliac lymphadenopathy, with heterogenous right and left inguinal malignant masses; positron emission tomography (PET) scan showed activity; then the right inguinal lymph node fine-needle aspiration (FNA) followed by an excisional biopsy confirmed CLL/SLL. Results of the initial lab work are presented in Tables 1-3.

**Table 1:** Complete blood count

Lab	Value	Reference Range
White blood cells	6.7	3.8-10.6 K/uL
Hemoglobin	12.8	12-15 g/dL
Platelets	258	150-450 K/uL

**Table 2:** Complete metabolic panel

Lab	Value	Reference Range
Glucose	81	60-140 mg/dL
Sodium	145	135-145 mmol/L
Potassium	4.5	3.5-5.0 mmol/L
Total protein	5.9	6.0-8.3 g/dL
Albumin	4.2	3.7-4.8 g/dL
Globulin	1.7	2.5-4.1 g/dL
Blood urea nitrogen	20	10-25 mg/dL
Creatinine	0.67	<1.03 mg/dL
Lactate dehydrogenase (LDH)	332	140-280 U/L
Aspartate aminotransferase (AST)	23	<35 lU/L
Alanine aminotransferase (ALT)	22	<40 lU/L

**Table 3:** Inflammatory and fungal markers

Lab	Value	Reference Range
Erythrocyte sedimentation rate (ESR)	102	<15 mm/h
C-reactive protein (CRP)	70.4	<10 mg/L
Fungitell	<31	<60 pg/mL

Given that the patient had localized disease and was asymptomatic, hematology recommended conservative management and forgo starting chemotherapy. Six weeks later, she complained of left groin pain and erythema with enlargement of underlying left inguinal lymph node, followed by drainage of purulent discharge associated with subjective fevers, night sweats, chills, loss of weight, drenching sweats, and failed a course of antibiotic therapy.

A left inguinal lymph node biopsy was obtained to exclude transformation to highgrade lymphoma, which revealed necrotizing granuloma, B-cell CLL/SLL, and absence of Richter's transformation. As the patient continued to be symptomatic with fever and chills, she was referred to infectious disease (ID) to investigate for possible atypical infection. Further workup revealed positive Epstein-Barr virus (EBV) serology. Pathology was contacted for possible reexamination of the lymph node (LN) biopsy for EBV infection, which was not seen. However, there was evidence of viral inclusions concerning for herpes simplex virus (HSV) infection. Subsequently, the patient was started on antiviral therapy with a good response.

The patient received valacyclovir for four weeks with an improvement in her symptoms. As the patient has CLL and hypogammaglobulinemia (immunocompromised), lifelong antiviral suppression was advised; however, the patient refused to be on long-term therapy.

Pathology

A right inguinal lymph node excisional biopsy showed proliferation of neoplastic small lymphocytes. The lab results were positive for CD20, CD5, and B-cell lymphoma 2 (BCL-2) and negative for CD3, CD10, BCL-6, cyclin D1, and multiple myeloma 1 (Mum1). There was no evidence of large cell transformation/diffuse large B-cell lymphoma, and no necrosis was found. Molecular testing was negative for P53 sequencing. A left inguinal lymph node pathology showed necrotizing granulomatous inflammation; however special stains were negative for acid-fast bacilli (AFB), fungal organism, accompanying residual CLL/SLL and Richter’s transformation.

Pathology reexamination of the left inguinal lymph node showed that within one section at a 1-mm focus of the purulent debris and granulomatous inflammation, there were HSV viral inclusions, which were initially interpreted as necrotic degenerative changes. There was no evidence of transformation to a necrotic diffuse large B-cell lymphoma on CD3, CD20, and CD30 stains. The in situ hybridization with appropriate controls for Epstein-Barr virus-encoded RNA was negative.

**Figure 1:**
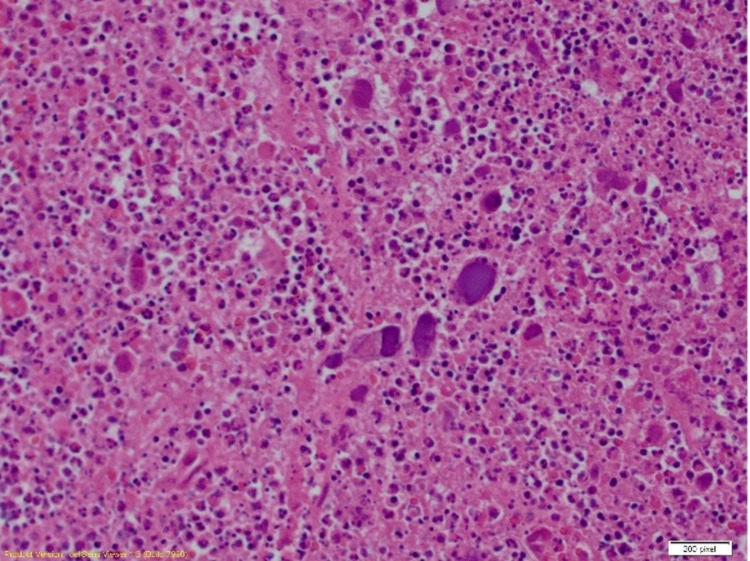
Excision of the lymph node revealed necrotizing granulomatous inflammation (left side of the image) with accompanying residual chronic lymphocytic leukemia/small lymphocytic lymphoma (right side of the image) (100x) (immunohistochemical staining) Image credit: Henry Ford Health System, Department of Pathology, Jackson, USA.

**Figure 2:**
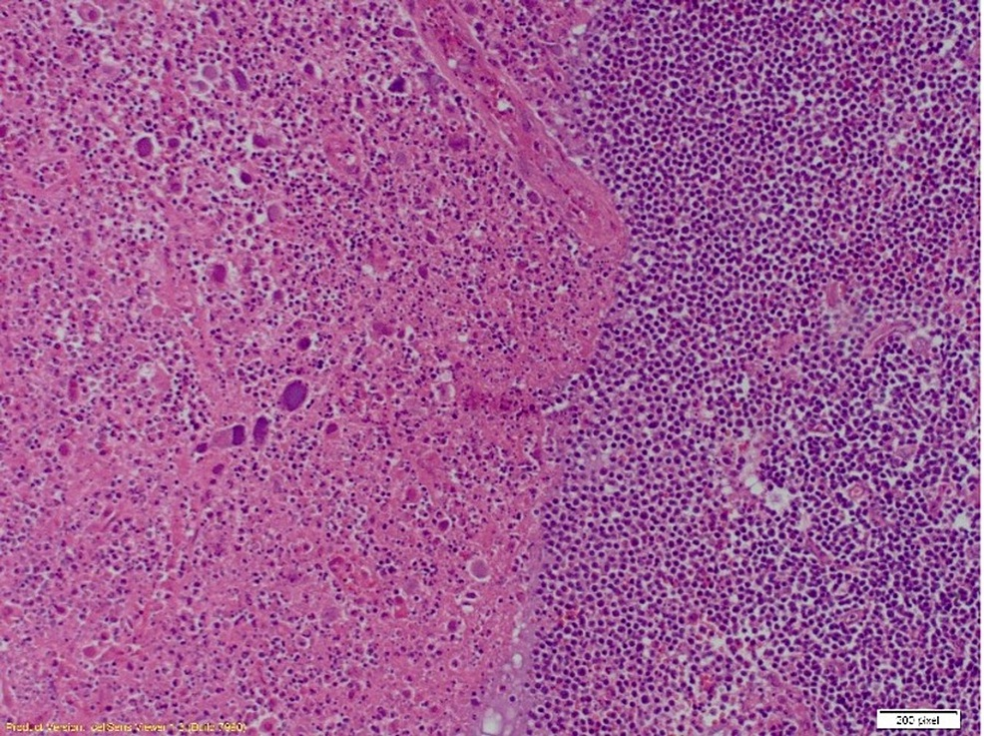
Excision of the lymph node revealed within the purulent debris and granulomatous inflammation. There are viral inclusions with morphological characteristics of herpes simplex virus (200x) (immunohistochemical staining) Image credit: Henry Ford Health System, Department of Pathology, Jackson, USA.

As morphologically suggested, immunohistochemistry for a cocktail including HSV-1 and HSV-2 was positive. This confirms HSV necrotizing lymphadenitis. Results of the HSV viral panel are presented in Table 4, and images of the pathology slides are shown in Figures 1, 2.

**Table 4:** Viral panel HSV: Herpes simplex virus.

Lab	Result
HSV1	Not detected
HSV2	Detected

## Discussion

Granulomatous lymphadenitis (GLA) can be classified as noninfectious GLA and infectious GLA. Noninfectious GLA includes sarcoidosis and sarcoid-like reaction. Infectious GLA can be classified further as suppurative lymphadenitis (examples include tularemia, cat scratch disease, fungal infection, etc.) and nonsuppurative lymphadenitis (examples include tuberculosis, toxoplasmosis, syphilis, etc.) [7]. Viral lymphadenitis is very rare, and common pathogens include human immunodeficiency virus, EBV, and HSV.

There are very few documented cases of HSV lymphadenitis in patients with CLL [6,8]. The isolated involvement of lymph nodes emphasizes the importance of sufficient tissue sampling and thorough tissue examination for detecting the eliciting agent. In this case, a fine-needle biopsy was taken, and a more thorough reexamination of the biopsy sample showed viral inclusion bodies suggesting HSV histology. This prompted the detection of HSV in blood via polymerase chain reaction (PCR). Immunohistochemistry was also positive.

The patient’s lymphadenitis persisted and worsened over a few months. Diagnosis on initial presentation was missed due to a lack of pathological evidence. Since the patient had a diagnosis of CLL, initial FNA was done to exclude transformation to high-grade lymphoma, which it did. It also showed necrotizing granuloma, BCLL/SLL, but no Richter's transformation. Due to the persistence of the patient’s symptoms such as fevers and chills, a possible infectious etiology was considered. Reexamination of the sample to look for viral infectious etiology made a diagnosis of HSV lymphadenitis. PCR is currently considered the most sensitive test for detecting HSV, and workup should include HSV PCR testing where it is suspected.

As described before, biopsy-proven HSV lymphadenitis is extremely rare. An observational study from the Mayo Clinic, Rochester, USA, examined 286 patients who had undergone lymph node biopsy for rapidly progressive lymphadenopathy from a total of 3,040 lymph node biopsies from CLL patients between 2003 and 2012. It was found that 3.9% of the patients had infectious lymphadenitis, and only three patients had HSV lymphadenitis [6].

The patient was treated with valacyclovir for four weeks with an improvement in her symptoms. As the patient has CLL (immunocompromised) [9], lifelong antiviral suppression was advised, but she refused the therapy. Currently, there are no guidelines for the treatment of HSV lymphadenitis. Some cases have described a few antiviral regimens, while others have suggested that HSV lymphadenitis might be selflimited and may not require treatment. However, this case demonstrates the progression of HSV lymphadenitis when left undiagnosed and untreated, thereby highlighting the importance of accurate diagnosis and appropriate treatment to prevent its progression. This case also brings forward the importance of consideration of viral infections in patients with suspected lymphadenitis. This holds true, especially in immunocompromised patients.

Necrotizing lymphadenitis in patients with CLL raises the suspicion of Richter’s transformation (RT), yet clinicians also should be aware of HSV infection [10]. Patients with CLL/SLL have impaired innate and adaptive immune responses with T-cell dysfunction, low serum complements, hypogammaglobulinemia, and inefficient antibody response, which explains the increased risk of some patients to fulminant infection even before chemotherapy.

## Conclusions

Necrotizing granulomatous lymphadenitis due to HSV infection is extremely rare, with only a few cases reported in the literature. HSV infection (often reactivation) of lymph nodes in patients with CLL/SLL results in clinicopathological and radiological findings that might be confusing with Richter's transformation, which makes the diagnosis slightly challenging. However, because Richter’s transformation is associated with a dismal outcome, efforts should be made to differentiate both diseases. HSV-induced necrotizing granulomatous lymphadenitis is a self-limited disease and has a better response to antiviral therapy.
